# Emerging Role of Pericytes and Their Secretome in the Heart

**DOI:** 10.3390/cells10030548

**Published:** 2021-03-04

**Authors:** Han Su, Aubrey C. Cantrell, Heng Zeng, Shai-Hong Zhu, Jian-Xiong Chen

**Affiliations:** 1 Department of Pharmacology and Toxicology, University of Mississippi Medical Center, Jackson, MS 39216, USA; suhan168302050@gmail.com (H.S.); acantrell@umc.edu (A.C.C.); hzeng@umc.edu (H.Z.); 2 Department of General Surgery, Third Xiangya Hospital, Central South University, Changsha 410013, China; zshxy3yy@163.com

**Keywords:** pericytes, endothelial cells, VSMCs, cardiomyocytes, regeneration

## Abstract

Pericytes, as mural cells covering microvascular capillaries, play an essential role in vascular remodeling and maintaining vascular functions and blood flow. Pericytes are crucial participants in the physiological and pathological processes of cardiovascular disease. They actively interact with endothelial cells, vascular smooth muscle cells (VSMCs), fibroblasts, and other cells via the mechanisms involved in the secretome. The secretome of pericytes, along with diverse molecules including proinflammatory cytokines, angiogenic growth factors, and the extracellular matrix (ECM), has great impacts on the formation, stabilization, and remodeling of vasculature, as well as on regenerative processes. Emerging evidence also indicates that pericytes work as mesenchymal cells or progenitor cells in cardiovascular regeneration. Their capacity for differentiation also contributes to vascular remodeling in different ways. Previous studies primarily focused on the roles of pericytes in organs such as the brain, retina, lung, and kidney; very few studies have focused on pericytes in the heart. In this review, following a brief introduction of the origin and fundamental characteristics of pericytes, we focus on pericyte functions and mechanisms with respect to heart disease, ending with the promising use of cardiac pericytes in the treatment of ischemic heart failure.

## 1. Introduction

There is a gradual progress to understanding the essence of pericytes. Although pericytes were first described by Dr. Eberth [1], the comprehensive understanding of pericyte origin came from the study of Dr. Rouget [2]. In his study, the morphology of pericytes was demonstrated for the first time, showing them to be in close proximity to endothelial cells [3]. Later, some studies revealed the expression of actin and myosin in pericytes by immunocytochemistry, indicating that pericytes have contractile elements to regulate blood flow and vascular permeability [4,5,6]. Recently, it was found that pericytes work as potential progenitors, differentiating into other cell types under specific circumstances [7]. In recent years, the nature of pericytes was further characterized, and pericyte loss has been identified as a key contributor to human diseases, including diabetic retinopathy, Alzheimer’s disease, and pulmonary hypertension [1].

The current definition of pericytes is well accepted; they belong to mural cells and are found residing within the basement membrane in microvessels [8]. Pericytes cover and adhere to the surface of endothelial cells (ECs) in the microcirculation, including in terminal arterioles, precapillary venules, and capillaries [8]. Structural contacts and biological interactions occur frequently between these two types of cells, contributing to the formation, maintenance, and remodeling of vasculature [7]. Additionally, differentiation of pericytes gives rise to other type of cells, such as adipocytes, vascular smooth muscle cells (VSMCs), and myofibroblasts, and consequently modulates the vascular network and blood flow [1]. Furthermore, pericytes communicate and interact with adjacent cells to support the vasculature structurally and functionally [1].

In the present review, we focus on the fundamental characteristics of cardiac pericytes and their interactions with adjacent cells, as well as their role in heart failure.

## 2. Characteristics of Pericytes

### 2.1. Origin

A recent study indicated that human pluripotent stem cells (hPSCs) may be a viable source of pericytes in vitro. HPSCs first develop into mesenchymal progenitor cells, which differentiate into immature SMCs and immature pericytes [9]. The immature pericytes develop into two mature phenotypes (pericytes type-I and type-II) and are distributed to multiple organs and tissues, taking center stage in vasculature [9]. More specifically, a series of works by Birbrair et al. demonstrated several differences between pericytes type-I and type-II in terms of marker, location, and function (Table 1) [10,11,12].

During embryonic development, the majority of pericytes originate in the gut, liver, lung, and heart, following mesothelium–mural cell differentiation [1,13,14]. For example, pericytes in the heart originate from the epicardium surrounding the outer layer of the heart. During the growth of the heart, epicardial to mesenchymal transition (EMT) occurs, giving rise to mesenchymal cells [15]. These mesenchymal cells then develop into mural cells and fibroblasts in the heart. Other than the mesothelium, there are several cell types from which pericytes originate. A recent study revealed that endothelial cells may also work as progenitors of pericytes during development. In this study, endothelial–mesenchymal transition (End-MT) resulted in an increase in mesenchymal cells, which contribute to the development of pericytes [16]. In the central nervous system, the neural crest was identified to be the origin of pericytes [17,18].

### 2.2. Diversity of Pericytes

Although endothelia are surrounded by pericytes in morphology, only parts of ECs are covered by pericytes (PCs), and the PC/EC coverage rate varies in different tissues, ranging from 1% to 50%. This variation leads to a different degree of contribution by pericytes according to their location (Table 2) [19]. For example, the coverage of pericytes to endothelial cells in the heart is about 1:2 to 1:3 [20]. Pericytes in the central nervous system such as the blood–brain barrier (BBB) have the highest coverage, at about 1:1 [21]. The BBB is structurally and functionally dependent on the coverage of cerebral pericytes [21,22]. The higher density of pericytes in the BBB is imperative in maintaining the vascular integrity and function of the brain. Previous studies have shown that detachment of pericytes from endothelial cells results in an impairment of brain vascular integrity and BBB function [23]. In several organs such as skeletal muscle, the pericyte–endothelial cell ratio reaches as low as 1:100 [15]. Consequently, this lower pericyte density implies that pericytes are less important in these areas, making the vascular network more vulnerable [24]. In addition to variations in coverage, pericytes also display distinguished shapes depending on their location. For example, pericytes in the central nervous system are solitary and stellate-shaped, while cardiac pericytes were found to be spindle-shaped [19,25]. Meanwhile, pericytes in the kidney are rounded and compact and, thus, more regular than those of the central nervous system and the heart [19]. Although mural cells around microvessels are all defined as pericytes, their morphology, function, and distribution can vary widely [7,8,19,26]. This diversity might contribute to their stem-cell features.

### 2.3. Identification

The features of pericytes are not only distinguished by PC/EC coverage and pericyte shape, but also by numerous markers that verify the heterogeneity of pericytes. Some markers such as smooth muscle α-actin (α-SMA), cluster of differentiation 13 (CD13), desmin, NG-2, and platelet-derived growth factor receptor (PDGFR-β) are classic, being well recognized and widely used to represent pericytes [1]. Other markers such as the regulator of G protein signaling 5 (RGS 5), Endosialin, and delta-like homolog 1 (DLK-1) are newly identified markers for pericytes [1,19]. The variation of markers depends on the stage of the pericyte, as well as where it is located. For instance, α-SMA^+^ pericytes are usually found in the retina and retinopathy [27,28]. The expression of NG2 and PDGFR-β was found to be upregulated in the pericytes of tumors and cardiovascular disease [1,29]. Additionally, increased levels of RGS5 are always observed in pericytes of angiogenesis, indicating the degree of vascular remodeling [30]. Moreover, a previous study revealed that pericyte type-I is a capillary phenotype, which can be differentiated from pericyte type-II as an arteriolar phenotype, thus indicating distinguished functions [9].

Despite the diversity of these markers, none of them can definitively indicate pericytes. This is due to pericyte properties as potential progenitor cells. Pericytes also share common markers with other adjacent cells. PDGFR-β, a well-known marker for pericytes, is not only expressed in pericytes but also in SMCs, myofibroblasts, mesenchymal stem cells, and neuronal progenitors [31]. Another classic pericyte marker, NG2, can be found in various cell types such as adult skin stem cells, adipocytes, VSMCs, and oligodendrocyte progenitors [32,33]. Additionally, α-SMA is the common marker for contractile pericytes, and it is also expressed in VSMCs and myofibroblasts [34]. Currently, there are no specific markers that could identify pericytes, making tracing pericytes with these markers less reliable.

This limitation also applies to tracing cardiac pericytes. Pericytes in the heart are usually marked with α-SMA, NG-2, and PDGFR-β [20]. In recent years, efforts have been made by many studies to develop a better model for tracing cardiac pericytes specifically. Using PDGFR-β-Cre mice, a study revealed that PDGFR-β is not suitable for representing pericytes because PDGFR-β^+^ pericytes fail to behave as progenitor cells [35]. Using NG2-DsRed mice as a practical animal model to trace pericytes, accumulating evidence indicates a crucial role played by NG2^+^ pericytes in cardiovascular disease. Using NG2-DsRed mice, Volz et al. reported that pericytes derived from the epicardium were the origin of VSMCs in the coronary artery [33]. Using NG2-DsRed mice, we demonstrated an important contribution of NG2 pericytes to myofibroblast transition in angiotensin-II-induced cardiac fibrosis and renal fibrosis [29,36,37]. In line with these studies, NG2 pericytes were also proven to be beneficial for BBB function and vascular permeability in NG2-DsRed mice [38]. Therefore, NG2-DsRed mice may be considered as a specific model for tracing cardiac NG2 pericytes. More studies are warranted to investigate the role of NG2^+^ pericytes in hypertension and hypertensive heart failure using NG2-DsRed mice.

### 2.4. The Secretome of Pericytes

The secretory capability of pericytes is being explored, and the potential implications for tissue regeneration are beginning to emerge. Various cytokines and factors such as immune-regulatory factors, angiogenic growth factors, quiescence-inducing factors, and the extracellular matrix (ECM) have been reported to be released by pericytes, which modulate a series of physiological and pathophysiological processes, especially in tissue repair and regeneration [39,40].

Under basal conditions, several proinflammatory factors such as interleukin-6, 8 (IL-6, 8), tumor necrosis factor alpha (TNF-α), interferon gamma-induced protein 10 (IP-10), and adhesion molecules have been found in pericytes, contributing to the activities of T-cells [41,42,43]. Under the stimulation of several pathogens such as lipopolysaccharide (LPS), granulocyte colony, and high glucose, cytokines such as Eotaxin and Rantes are produced by pericytes, resulting in the exacerbation of tissue injury [44]. Interestingly, several anti-inflammatory factors are also reported to be secreted by pericytes. For instance, leukemia inhibitory factor (LIF), cyclooxygenase-2 (COX-2), and heme oxygenase-1 (HMOX-1) were secreted from pericytes and worked as inhibitory cytokines during inflammation [45,46]. Moreover, pericytes induce stem-cell quiescence and protect them from exhaustion and senescence by releasing quiescence-inducing factors such as bone morphogenetic protein-4, 6, 7 (Bmp-4, 6, 7), which preserves stem-cell regenerative capabilities [47,48].

Angiogenesis is essential for tissue regeneration and repair since more oxygen and nutrients can be distributed with formation of neovessels. Angiogenic growth factors such as transforming growth factor-β (TGF-β), angiopoietins-1 (Ang-1), and vascular endothelial growth factor (VEGF) from pericytes are responsible for differentiation and proliferation of both pericytes and endothelial cells, aiding in the formation and stabilization of neovessels [1,19,49]. Additionally, the ECM secreted by pericytes plays an important role during tissue repair and regeneration [50,51]. In addition to the ECM, ECM-associated factors such as secreted protein acidic and cysteine-rich (SPARC), which regulates ECM formation and angiogenesis, are also found in pericytes [52]. Taken together, pericytes could secrete a large panel of cytokines and angiogenic molecules and growth factors, indicating that pericytes are a potential new therapeutic target for maintaining function or restoring damaged tissues and organs (Table 3).

## 3. Pericytes Cross-Talking with Adjacent Cells

### 3.1. Endothelial Cells

Pericytes are vascular mural cells, adhering to the surface of endothelial cells and becoming embedded within the vascular basement membrane. They usually emerge around microvessels such as precapillary arterioles, venules, and capillaries [53]. Without coverage of VSMCs, pericytes are the main mural cells in the microcirculation, and their reciprocal interactions with endothelial cells take center stage in the formation, stabilization, and remodeling of microvasculature, which consequently regulates capillary blood flow and vascular function [7].

#### 3.1.1. Reciprocal Interactions in Structures and Functions

In general, endothelial–pericyte communications are mediated through specialized intercellular junctions [19,54,55,56]. The peg–socket pocket is formed by cytoplasmic fingers and is one of the junctions known to combine endothelial cells and pericytes in structure. It contains adherent junctions and gap junctions responsible for connecting the cytoskeleton and cytoplasm of endothelial cells and pericytes [19,57].

**Figure 1:**
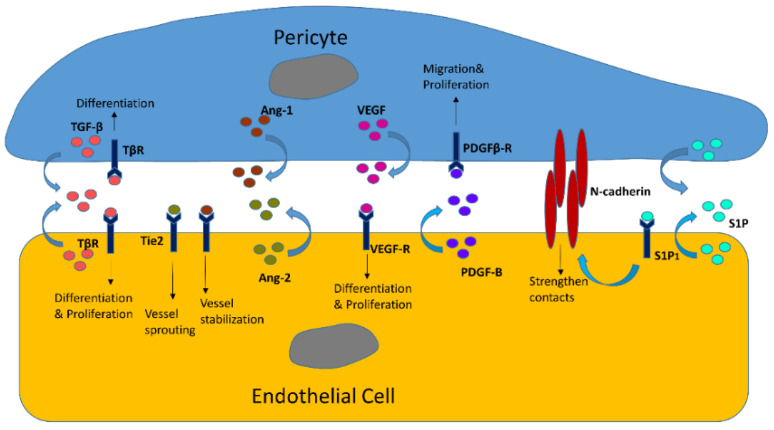
Interactions between endothelial cells and pericytes via secretory actions. (**1**) TGF-β could be produced by both pericytes and endothelial cells, mainly mediating differentiation of pericytes and endothelial cells. It also activates proliferation of endothelial cells; (**2**) angiopoietin-1 (Ang-1) from PCs and angiopoietin-2 (Ang-2) from ECs could combine Tie-2 in ECs (although a low level of Tie2 expression has been detected in pericytes, only a mild physiological phenotype was found after deletion of Tie-2 in PCs. The role of Ang-1/Tie-2 signal in pericytes still remains controversial); (**3**) VEGF, mainly derived from mesenchymal cells (pericytes), is responsible for differentiation into pericytes. Similar to TGF-β, it could also trigger proliferation of ECs. VEGF ligands include VEGF-A, B, C, D, E and receptors mainly include VEGFR1 and VEGFR2. Normally, VEGFR1 binds to VEGF-A and B, while VEGFR2 binds to VEGF-A, C, D, and E; (**4**) PDGF-B, derived from endothelial cells, is found in angiogenic sprouts and remodeling arteries. It triggers migration and proliferation of pericytes via combination with PDGFR-β; (**5**) sphingosine-1-phosphate (S1P) is expressed in endothelial cells. Its signaling pathway strengthens the contacts between endothelial cells and N-cadherin, which induces migration of pericytes toward endothelial cells and further stabilizes the combination of two cells.

The interactions between pericytes and endothelial cells is evident not only by structure but also by function. On one hand, proliferation, differentiation, contractility, and stabilization of endothelial cells may be mediated by pericytes. During angiogenesis, a series of growth factors from pericytes such as TGF-β and VEGF stimulate the proliferation and transition of endothelial cells [58,59]. The immature basement together with proliferative endothelial cells may be stabilized by the recruitment of pericytes, building up a foundation for more proliferative endothelium [8]. Conversely, endothelial cells could also influence pericytes. Studies show that several cytokines from the endothelium may have an important role in the movement of pericytes. For instance, loss of endothelial PDGF-BB and WNT5a leads to decreased motility of pericytes and, thus, less pericyte movement toward endothelial cells [60]. Disturbance of VEGF-A could prevent the recruitment and migration of pericytes [53]. These alterations cause reduced pericyte recruitment and inhibition of angiogenesis, indicating the importance of endothelial cells for pericytes in vascular regeneration. The communication of pericytes and endothelial cells weighs importantly in the microvasculature. Overall, endothelial cells are the primary target for pericytes, and their reciprocal communications are essential for neovascularization, maintaining vascular stabilization, and other important physiological processes in the vascular network. Several crucial factors such as TGF-β, angiopoietins/Tie-2, PDGF, VEGF, and sphingosine-1-phosphate receptor (S1P1) are involved in the interactions between endothelial cells and pericytes (Figure 1) [58,59,61,62,63].

#### 3.1.2. EC/Pericyte Interactions in the Heart

Pericytes make direct contacts with blood vessel endothelia, as they are embedded in the basement membrane of the microvasculature [19,64]. There is an abundance of pericytes in the myocardial capillary of the human heart. Pericyte coverage is associated with two or three ECs in the human heart [20]. Loss of pericytes has been shown to contribute to diabetic retinopathy and vascular leakage in diabetes [65,66,67,68]. Additionally, microvascular dysfunction in other vascular beds has been attributed to pericyte dysfunction [69]. For example, capillary pericytes have been shown to play a critical role in the regulation of cerebral blood flow during ischemia/reperfusion injury and the no-flow phenomenon in reperfusion [70,71]. Despite the fact that pericytes are the second most abundant non-cardiomyocyte cells in the heart, after ECs [20,69,70,72,73,74], little is known about the role of cardiac pericytes in the regulation of coronary blood flow and heart failure. Treatment of mice with sunitinib malate was shown to disrupt EC/pericyte interactions and lead to impaired coronary blood flow (CBF) and cardiac dysfunction [69]. Notch3 plays a critical regulatory role in pericyte differentiation and recruitment by regulating pericyte numbers and maintaining vascular integrity during development [75,76,77,78]. Notch3 knockout (KO) mice challenged with angiotensin-II were shown to develop coronary microvascular dysfunction and heart failure [79,80]. We demonstrated that a significant reduction in pericytes in the mouse heart resulted from knockout of Notch3, leading to impairments of pericyte/EC coverage and coronary flow reserve (CFR). Furthermore, a larger infarcted size and higher mortality in mice were observed, suggesting that knockout of Notch3 sensitized the heart to ischemic injury [81]. Additionally, Notch3 deficiency resulted in a reduced number of NG2^+^ (pericyte)/Sca1^+^/c-kit^+^ progenitor cells and impaired microvascular stabilization, thus promoting microvascular leakage and inflammation in ischemic hearts [81]. Our study strongly suggests that, in response to myocardial ischemia, both the maturation and the integrity of the coronary microvasculature requires the presence of cardiac pericytes. Cardiac pericytes show a very promising therapeutic potential with regard to coronary no-reflow and heart failure [81,82]. Furthermore, Notch3 may prove to be a novel therapeutic target for cardiac pericyte–myofibroblast transition and coronary no-reflow after ischemia/reperfusion.

We also showed that severe impairment of pericyte/EC coverage is found in the hearts of obese mice [83]. Following knockout of Sirtuin3 (SIRT3) in mice, reduced pericyte/EC coverage was observed in the heart, along with a significant reduction of CFR [84]. Furthermore, our study indicated that pericyte loss in SIRT3 KO mice may be partially attributed to impairment of the angiopoietins/Tie-2 and hypoxia-inducible factor (HIF)-2α/Notch3 signaling pathways [85]. Although the exact mechanism of pericyte loss was not investigated, studies have shown that loss of pericytes or detachment of pericytes from the capillary may result in differentiation of pericytes into myofibroblasts. This may be a contributing factor in the deposition of excessive fibrosis and myocardial stiffness, which in turn may contribute to heart failure [86,87]. Therefore, we hypothesize that disruption of SIRT3 signaling may cause disruption of endothelial cell/pericyte communications and result in pericyte detachment, leading to pericyte–fibroblast transition and resulting in hypertensive- or diabetes-associated myocardial and vascular stiffness [88].

### 3.2. Vascular Smooth Muscle Cells (VSMCs)

Vascular smooth muscle cells are another type of mural cells that surround vessels, regulating vascular tone and mediating vascular remodeling. Pericytes usually encircle microvessels such as capillaries and come into direct contact with endothelial cells. Meanwhile, VSMCs are mainly located around the vascular wall of large blood vessels such as arteries and veins and do not contact the endothelium directly [89]. It seems that pericytes and VSMCs are responsible for the different functions in different vessels and areas; however, very close connections are found between pericytes and VSMCs. Not only do they share the same origin, but they also express a series of common markers such as PDGFR-β, NG2, α-SMA, CD13, and Desmin [1]. More investigations are needed to clarify the interactions and differences between pericytes and VSMCs.

#### 3.2.1. Pericyte Coordination with VSMCs

Both pericytes and VSMCs, as vascular mural cells, embrace vessels and are involved in the fabrication of vascular structure with the assistance of newly formed fibrin microfibers and endothelial cells, thus enhancing the stabilization and sustaining functions of the vascular network [90]. It is reported that the trilayer vascular graft, including fibroblasts, pericytes, VSMCs, and endothelial cells, supports vascular structure and acts as a barrier for protection [91]. Additionally, endothelial cells are thought to be a common target for these two cells in order to benefit vascular function. Proliferation of endothelial cells induced by pericyte–endothelial cell interactions could improve the contractility of vessels and reduce vascular leakage [89]. VSMC–endothelial cell interactions have been shown to be responsible for the stabilization of nascent vessels through secreting the extracellular matrix [92]. As either pericytes or VSMCs, mural cells play an imperative role in supporting vascular structure and fabrication of new vessels.

#### 3.2.2. SMC-Like Properties of Pericytes

Similar to SMCs, pericytes also express α-SMA, indicating SMC-like properties of pericytes in microvessels [70,93,94]. Previous studies revealed that the contractility of α-SMA^+^ pericytes contributes to restriction of blood flow in the brain, retina, and pancreas [6,95,96]. In line with these findings, cardiac pericytes were found to express actin and myosin in the heart [6]. Increased levels of cellular Ca^2+^ lead to contraction of pericytes, induction of extension of actomyosin-containing processes, and reduction in capillary diameter. The reduction in capillary diameter further induces restriction of blood flow in the microcirculation [93]. Contrarily, one study showed that, in addition to the compression from cardiomyocytes and endothelial cells and the adherence of leukocytes, disruption of pericyte–capillary interactions by PDGF-B/PDGFR-β suppresses the contractility of pericytes, leading to a significant increase in blood flow [94]. Furthermore, treatment with adenosine for relaxation of pericytes reduces the contractility of pericytes and significantly improves blood flow in the heart [94]. These studies provide strong evidence that pericytes function as SMC-like cells, and that contractility of pericytes could influence blood flow via restriction of capillaries.

#### 3.2.3. Pericyte–VSMC Transition

As described above, the expression of α-SMA in pericytes could be explained by the notion that pericytes shared progenitors with SMCs or their SMC-like features. Additionally, the expression of α-SMA in pericytes may be due to pericyte differentiation to VSMCs [97,98,99]. Though endothelial cells are usually characterized as the main source of VSMCs, pericytes, and other cells around vessels [9], pericytes derived from endothelial intermediates or mesenchymal progenitors are also known for their role as potential progenitor cells [99]. Pericytes are able to differentiate into a variety of cell types such as osteoblasts, adipocytes, fibroblasts, chondrocytes, and VSMCs [97,98,99]. It has been reported that epicardium-derived pericytes can differentiate into VSMCs in the heart [36]. Moreover, epicardium-derived pericytes marked solely with PDGFR-β^+^/Notch3^+^/NG2^+^/PDGFR-α^−^ were observed and wrapped in the coronary artery remodeling (CA) zone, further supporting the role of pericytes as an origin of coronary VSMCs [36,100,101]. Our recent study also showed a co-staining of NG2-DsRed and α-SMA cells in the mouse coronary artery, indicating the potential transition from pericytes to VSMCs [29]. In addition, TGF-β secreted by pericytes may contribute to pericyte–VSMC transition [1]. These studies provide evidence that pericytes could differentiate into VSMCs and be the therapeutic target for coronary remodeling.

### 3.3. Pericyte Reciprocal Interactions with Other Cells

**Figure 2:**
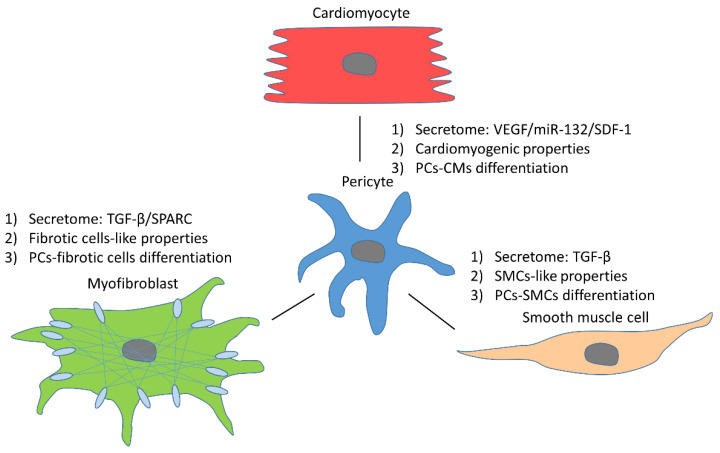
Interactions and differentiation between pericytes and their adjacent cells. (**1**) Cardiomyocytes. Through secreting VEGF and miR-132, pericytes could induce survival responses in cardiomyocytes. Moreover, stromal cell-derived factor-1 (SDF-1) from pericytes activates the mobilization of stem cells, benefiting cardiomyogenesis. Furthermore, some pericytes, sharing the same origin of epicardium with cardiomyocytes, were found to function as cardiomyocytes because of their myogenic capacity. Lastly, some immature cardiomyocytes are differentiated from pericytes. (**2**) VSMCs. Pericytes could produce TGF-β that is responsible for the differentiation and proliferation of VSMCs. In addition, some pericytes also show SMCs-like properties due to expression of actin and myosin (α-SMA). Likewise, because of the PC–SMC transition, pericytes can be defined as one main source of VSMCs. (**3**) Myofibroblasts. Pericyte-derived TGF-β and SPARC could regulate the ECM. Other than enhancing the ECM via the secretome, type-I pericytes could produce the ECM directly. Moreover, PC–myofibroblast differentiation contributes to fibrogenesis.

In addition to endothelial cells and VSMCs, pericytes are also associated with or interact with other adjacent cell types such as adipocytes, cardiomyocytes, and fibrotic cells. The interactions between pericytes and other cells in the heart play an essential role in cardiac physiology and pathology (Figure 2) [102].

#### 3.3.1. Cardiomyocytes

Pericytes have an important function in cardiac remodeling and recovery from heart ischemia [103]. Similar to the endothelium, pericytes also play a supporting role in cardiomyocytes through the secretome. This was evidenced by the higher growth rate of cardiomyocytes cocultured with pericytes as compared to the control group without pericytes [104,105]. Several molecules secreted by pericytes were found to protect cardiomyocytes. For instance, VEGF and miR-132 from pericytes could induce survival responses via activation of the p-Akt pathway in cardiomyocytes [104]. Stromal cell-derived factor-1 (SDF-1), a molecule responsible for the mobilization and homing of stem cells, was found to be expressed in pericytes, which is beneficial for cardiomyogenesis [106,107]. In addition to having direct effects on cardiomyocytes, pericyte-mediated neovessel formation via release of angiogenic factors in the injured area is also beneficial for cardiac recovery by providing more nutrients and oxygen [108]. Furthermore, pericytes can induce compaction of fibrin gel, which is helpful for contractile forces of the heart, thus reducing the burden of cardiomyocytes [109,110].

In addition to supporting cardiomyocytes, some pericytes have been reported to function as cardiomyocytes, replacing cardiomyocytes and improving cardiac function. A study revealed that the common origin of cardiac progenitors and pericytes is the epicardium, suggesting the possibility that pericytes and cardiomyocytes share similar features [111,112]. In line with this finding, cardiac mesenchymal stem cells were found to express pericyte markers NG-2 and PDGFR-β, indicating that cardiomyogenic cells exhibit some properties of cardiac pericytes [112]. Some heart pericytes (hPCs) were reported to have myogenic capacity, as evidenced by expression of α-SMA and cardiac troponin-T (cTn-T) and exhibition of contractile features similar to those of cardiomyocytes [113]. Furthermore, pericytes in the heart are capable of differentiating into cardiomyocyte-like cells via miR-132 both in vivo and in vitro [89,104]. The existence of spontaneous calcium oscillations after co-culture of pericytes and cardiomyocytes further supports the role of pericytes as cardiomyocyte progenitor cells [112,113]. Keep in mind that cardiomyocytes differentiated from pericytes still retain an immature status. Further activation is needed to develop these newly formed cardiomyocytes into the mature phenotype [113].

Taken together, cardiac pericytes, through either cardiomyocyte-like features or transition to cardiomyocytes, are critical to sustain cardiac function during myocardial injury, implicating the crucial role of cardiac pericytes in cardiovascular regenerative medicine.

#### 3.3.2. Fibrotic Cells

Fibrosis is characterized by increased numbers of fibrotic cells and accumulation of the ECM. Fibrosis, presented in the heart or other organs such as the liver and kidney, belongs to a reparative process during tissue injury or adverse remodeling [114,115,116]. For instance, myocardial injury such as myocardial infarction and ischemia would lead to inflammatory responses, initiating cardiac fibrosis for replacement of these injured or dead cardiomyocytes [117]. Pericytes are one of the major players for the development of cardiac fibrosis via the secretome involved in producing the ECM and interactions with fibrotic cells.

Fibrotic cells include fibroblasts and myofibroblasts. The proliferation of fibroblasts and their trans-differentiation to myofibroblasts result in pathological fibrosis in the heart, and they are closely related to cardiac pericytes [118]. Several cytokines secreted by pericytes could induce the proliferation and activation of fibrotic cells. In recent studies, we found that NG2-DsRed^+^/TGF-β^+^ double-positive cells were abundant in the fibrotic heart and kidney in response to angiotensin-II-induced hypertension, further validating the role of pericytes in TGF-β production and fibrosis [29,37]. It was reported that pericyte-derived TGF-β is an essential factor for fibrosis development via secretion of the ECM and activation and proliferation of fibrotic cells [119].

In addition to direct interactions with fibrotic cells, pericytes could also promote fibrosis by functioning as fibrotic cells. The ECM, consisting of collagen, elastic fiber, and proteoglycans, is the major component of fibrosis [120]. It is well known that the ECM is mostly produced by myofibroblasts. However, a recent study suggested that type 1 pericytes could secret collagen, thus playing a role similar to that of myofibroblasts [121]. Via co-staining of NG2-DsRed and collagen I, we found NG2-DsRed^+^/collagen I^+^ double-positive cells in the fibrotic mouse heart and kidney, indicating the involvement of pericytes in producing collagen I [29,37]. Other ECM components such as fibronectin, perlecan, and nidogen-1 were also found to be expressed by pericytes [122]. Secreted protein acidic and cysteine-rich (SPARC), a protein responsible for ECM modulation, was also found to be secreted by pericytes in a recent study [52]. Furthermore, α-SMA, PDGFR-β, and endosialin are common markers for both pericytes and fibrotic cells, indicating the fibrotic properties of pericytes [1]. The similarities between pericytes and fibrotic cells make pericytes a critical contributor to the development of fibrosis.

Furthermore, previous studies showed that pericytes are progenitor cells that are able to differentiate into fibroblasts and myofibroblasts, which are activated by growth factors from pericytes such as TGF-β and PDGFR-β [116,120]. Using NG2-DsRed mice with a specific marker for pericytes, we observed DsRed^+^/FSP-1^+^ and DsRed^+^/α-SMA^+^ double-positive cells in heart tissues, indicating differentiation of cardiac pericytes to fibroblasts and myofibroblasts [29].

Interactions with fibrotic cells, replacement of fibrotic cells, and differentiation into fibrotic cells make pericytes a key player in the physiological and pathological processes of fibrosis. However, the roles of pericytes in fibrosis under some specific conditions are controversial. Previous studies demonstrated that pericytes reduce fibrosis via inhibiting proliferation of fibroblasts and differentiation into myofibroblasts in the infarcted heart [123,124]. More works need to be done to further clarify the roles of pericytes in cardiac fibrosis.

#### 3.3.3. Telocytes

Telocytes, distributed in almost all organs, are described as a special type of interstitial cells recognized by telopodes (long, thin, and moniliform) [125,126]. With their long cytoplasmic processes, telocytes could form three-dimentional networks and get involved in heterocellular contacts with adjacent cells including cardiomyocytes, endothelia, and pericytes [127]. A previous study showed telocyte–capillary junctions through a telescope [128]. Together with endothelia and pericytes, cardiac telocytes play an essential role in integrating all the information in the vascular system.

Interestingly, telocytes are tightly associated with pericytes. A series of markers such as PDGFR-α, PDGFR-β, and vimentin are expressed in both telocytes and pericytes. Furthermore, it is relatively confusing when distinguishing pericytes from telocytes in two-dimensional cuts because of their similar look [126]. In addition, it was proposed that telocytes could originate from the process of endocardial-to-pericyte transformation [129]. These data strongly indicate a relationship; however, more exploration is needed for further validation.

## 4. Pericytes and Blood Flow

As mural cells around vessels, pericytes display a series of features such as contractility, differentiation ability, and communication with endothelial cells, which are tightly associated with blood flow in different ways.

### 4.1. Pericytes and VSMCs

Pericytes and VSMCs are both located around vessels and are unique perivascular mural cells in these areas. Moreover, pericytes have the same origin as VSMCs and express α-SMA, desmin, and myosin, suggesting VSMC-like properties of pericytes. These SMC-like properties of pericytes could function in mediating contractility and dilation of microcirculation, suggesting the pericyte as an important regulator of blood flow [6,74,95,130,131]. It has been reported that loss of or reduction in pericyte coverage in capillaries in islets leads to a disruption of blood flow, resulting in impairment of glucose tolerance and islet function in obesity and diabetes [96]. Additionally, loss of pericytes following treatment with sunitinib causes a reduction in coronary blood flow and impairment of coronary flow reserve (CFR) in mice [15]. During myocardial infarction, loss of pericytes results in no-reflow after reperfusion, thus exacerbating ischemic/reperfusion injuries and increasing incidence of death [74]. Increased oxidative stress and shortage of ATP during ischemia/reperfusion could further lead to the constriction of pericytes. These constricted pericytes narrow the capillaries and reduce blood flow, further worsening microvascular dysfunction and promoting heart failure [93,95,132]. Conversely, treatment with adenosine ameliorates coronary blood flow via relaxing pericytes [133]. These studies strongly suggest pericytes as a potential therapeutic target for improvement of microcirculation and blood flow.

As previously described, some pericytes may act as progenitor cells and possess the capacity to differentiate into other cell types such as VSMCs, cardiomyocytes, fibroblasts, myofibroblasts, and adipocytes [134]. Korn et al. observed the transition of pericytes to VSMCs in cerebral vessels during embryonic development [135]. In the heart, there were only a few studies focusing on the effects of pericytes on coronary blood flow. It was reported that the transition from cardiac pericytes into fibroblasts and myofibroblasts increases the pressure outside capillaries and results in microvascular rarefaction and impairments of CFR [29]. Using specific animal models to trace pericytes, epicardium-derived pericytes were proven to be progenitor cells of VSMCs in the coronary artery, thus affecting vascular tone [36].

### 4.2. Atherosclerosis

Coronary arterial disease (CAD), a leading cause of morbidity and death worldwide, could be a result of atherosclerosis [136]. Sub-endothelial intima is the location of the disturbance of blood flow, as well as the site of development of atherosclerosis [137]. The pathological process is defined as an interaction between sub-endothelial lipid overload and endothelial dysfunction, which leads to inflammatory responses in the vessel walls [137,138]. As discussed before, pericytes play a crucial role in angiogenesis, fibrosis, calcification, and inflammation in vessels. Furthermore, increases in pericytes and pericyte-like cells are always found in sites of atherosclerosis [139,140]. This evidence implies a tight correlation with the pathological process of atherosclerosis [131].

It is well documented that low-density lipoprotein (LDL) accumulation is one of the main reasons for the formation of atherosclerosis. In addition, inflammatory responses could be the starting point of LDL accumulation-induced atherosclerosis. For instance, expression of CD68, a scavenger receptor, could result in lipid accumulation, inducing thickening of vessel walls and the development of atherosclerosis [141]. As mentioned before, pericytes participate in inflammatory responses in vascular walls in many ways, such as differentiation to macrophages and secretion of proinflammatory cytokines [131]. Adverse conditions such as high glucose and reactive oxygen species could induce secretion of BMP-4 by pericytes in the vessel wall [142]. Likewise, under stimulation of proinflammatory cytokines (IL-17), pericytes could trigger neutrophil-mediated immunity via production of TNF-α, IL-6, 8 and other proinflammatory factors, playing a central part in the development of atherosclerosis [143]. Pericyte-induced inflammatory responses further exacerbate lipid accumulation in atherosclerotic vascular walls.

In large vessels, a proteoglycan-rich layer is located between the endothelium and muscular elastic layer, and it contains a series of cells such as pericytes [131]. Pericyte differentiation to osteogenic cells might contribute to the formation of maladaptive ectopic calcification [138,139,144]. The process of calcification accompanied by matrix remodeling also attributed to pericytes. Osteoprotegerin (OPG) could be secreted by pericytes and is associated with development of calcification [145]. All of these alterations are essential for the structure of atherosclerotic lesions. Furthermore, pericytes have a critical role in neovascularization in inflamed vascular walls, which promotes the formation of atherosclerosis [146]. Overall, through inflammatory responses, differentiation, and angiogenesis, pericytes contribute essentially to the development of atherosclerosis.

More specifically, when these pathological processes of atherosclerosis occur in the cardiac valve, corresponding impairments consequently emerge [147]. Among others, calcification of cardiac valve could be the most common damage caused by atherosclerosis [148]. Cardiac valves include the atrioventricular valve, aortic valve, and pulmonary valve, and they are responsible for regulating blood flow inside cardiac chambers. The calcification of these cardiac valves results in disturbance of blood flow and a heavier burden on the heart, which not only further develops it pathologically but also ultimately leads to cardiac dysfunction and heart failure [146,147]. Pericytes in cardiac valves retain the ability to differentiate into myofibroblasts and osteoblasts, causing fibrosis and calcification [142,145]. In contrast, pericytes also contribute to neovascularization in cardiac valves to increase delivery of oxygen and nutrients to these areas, which ameliorates the development of fibrosis and calcification in cardiac valves [61,149]. Thus, pericytes could have functions in both angiogenesis and calcification in stenosis of cardiac valves.

## 5. The Rapeutic Role of Pericytes in Infarcted Heart

**Figure 3:**
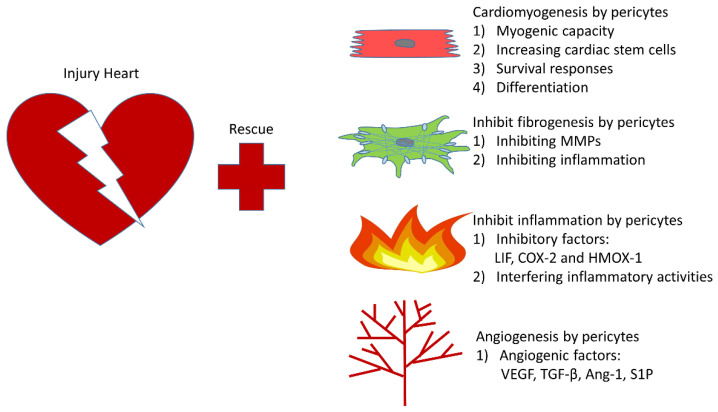
Therapeutic roles pericytes in infracted heart. (**1**) Cardiomyogenesis: a. pericytes could replace cardiomyocytes due to its myogenic capacity; b. pericytes could induce increases of cardiac stem cells and survival responses of cardiomyocytes; c. pericytes could increase cardiomyocyte differentiation. (**2**) Inhibition of fibrosis: a. pericytes inhibit the secretion of ECM and proliferation of fibrotic cells through MMPs; b. through inhibition of inflammation, pericytes could ameliorate myocardial fibrosis. (**3**) Suppression of inflammation: a. pericytes produce inhibitory factors such as LIF, COX-2 and HMOX-1 to attenuate inflammatory responses; b. by interfering with inflammatory activities of ECs, inflammation can be relieved. (**4**) Angiogenesis: through secreting pro-angiogenetic factors such as VEGF, TGF-β, Ang-1 and S1P, more vessels are formed to benefit the recovery.

Accumulating evidence indicates that loss of cardiac pericytes could lead to adverse effects via interference with myocardial blood flow. The disturbance of coronary/myocardial blood flow, due to pericyte-associated abnormalities such as stenosis of cardiac valves and no-reflow in capillaries, causes a reduction in blood supply to cardiomyocytes and exacerbates myocardial ischemia [94,149]. Pericytes are considered as protectors of the cardiac repair after myocardial infarction [72,113]. It was reported that transplanting human pericytes into an infarcted heart improved contractility of the injured heart [113]. Pericytes improve cardiac function and enhance cardiac repair after myocardial ischemia via attenuation of cardiac remodeling, alleviation of inflammatory responses, and induction of angiogenesis [150]. The potential mechanisms and therapeutic roles of PCs in ischemic heart have been summarized as followings (Figure 3):

### 5.1. Cardiomyogenesis

The cardiomyocyte is the major cell type in the heart. During myocardial infarction, the death of cardiomyocytes results in secondary responses including inflammation, fibrosis, and vascular leakage, consequently contributing to cardiac dysfunction [150]. Thus, our ultimate goal is to boost reparative cardiomyogenesis during cardiac repair [151]. Beltrami et al. revealed a cardiomyogenic phenotype of human heart pericytes (hHPs), as evidenced by the expression of cardiomyogenic transcription factors Nkx2.5 and GATA4 in vitro [112]. Interestingly, low levels of α-sarcomeric actinin (α-actinin) and cardiac myosin heavy chain, as well as no expression of cardiac troponin-T (cTn-T), are found in hHPs, indicating that hHPs may function as immature cardiomyocytes. Intriguingly, when coculturing hHPs with cardiomyocytes, mature cardiomyocyte markers such as cTn-T and atrial natriuretic peptide (ANP) are expressed in hHPs [113]. Consistent with this study, the potential role of pericytes as cardiomyocytes was also proven by Elisa et al. showing myogenic ability in type 2 pericytes [89]. Furthermore, rather than acting like cardiomyocytes, it was found that numbers of cardiac stem cells are significantly increased in the infarcted zone after pericyte transplantation, suggesting an essential role of pericytes in the source of cardiomyocytes [105]. On one hand, pericytes could improve cardiac repair via promoting the recruitment of cardiomyocytes. Through the Akt signaling pathway, pericytes activate the proliferation and survival signaling pathways of cardiomyocytes [104]. Additionally, SDF-1 secreted from pericytes may contribute to homing of cardiac stem cells [106]. On the other hand, pericytes can transition into cardiomyocytes directly to contribute to heart regeneration. TGF-β and miR-132, as molecules secreted by pericytes, are critical in this differentiation [104,117]. The differentiation and secretome of pericytes contribute to the reconstitution of major cell types and recovery of cardiac dysfunction after myocardial infarction. This evidence suggests pericytes as a potential therapeutic target for myocardial repair.

### 5.2. Cardiac Fibrosis

As previously described, a pericyte is defined as a contributor to fibrosis because of its ability to differentiate into fibrotic cells and secrete profibrotic cytokines. The contributions of pericytes to cardiac fibrosis in the infarcted heart are different than that during treatment with pericytes to alleviate cardiac dysfunction during myocardial infarction [123,124]. More specifically, matrix metalloproteinases (MMPs), as central mediators of fibrosis controlling extracellular matrix and proliferation of fibroblasts, were found to be reduced after the injection of pericytes, partly explaining the antifibrotic role of pericytes in infarcted heart [152,153]. Similarly, through MMP and endogenous inhibitors (TIMPs), transplantation of other mesenchymal stem cells also reduces cardiac fibrosis, further supporting the antifibrotic role of progenitor cells in the heart after myocardial infarction [153]. Additionally, inflammation is known to be a crucial factor for cardiac fibrosis and is regulated by pericytes [154,155,156]. Through interference with inflammatory responses, pericytes ameliorate cardiac fibrosis and improve cardiac function. Overall, the alleviation of fibrosis could reduce coronary stiffness and improve cardiac function, which is beneficial for regeneration after myocardial infarction [104,123].

### 5.3. Inflammatory Response

Necrosis is the main form of cell death in the infarcted heart, releasing intracellular contents and activating inflammatory responses [117]. The initiation of inflammatory response allows cleaning of dead cells and matrix debris, as well as the formation of scar tissue, benefiting the recovery of the infarcted heart [117]. Meanwhile, excessive infarction-induced inflammation after ischemia might lead to adverse remodeling, which further causes cardiac dysfunction. Mesenchymal stem cells with immunosuppressive function are evidenced by the suppression of T-lymphocyte and CD68^+^ phagocytic cells in the injured heart [45,105]. In line with these findings, injection of pericytes into the infarcted heart resulted in a reduction of monocyte/macrophage infiltration with reduced CD68 expression [123]. Emerging evidence suggests that the alleviation of inflammatory responses is attributed to paracrine cytokines from pericytes. Leukemia inhibitory factor (LIF), cyclooxygenase-2 (COX-2), and heme oxygenase-1 (HMOX-1) are among the anti-inflammatory factors produced by pericytes [45,46]. The secretion of these cytokines by pericytes contributes to these immunosuppressive effects in the infarcted heart. Furthermore, proinflammatory factors such as interleukin-1α (IL-1α), tumor necrosis factor-α (TNF-α), and interferon-γ (IFNγ) are found in pericytes at low levels, which is consistent with their inhibitory role in inflammation [46,157]. Furthermore, endothelial cells could synthesize and express chemokines such as vascular cell adhesion molecule-1 (VCAM-1) or intercellular cell adhesion molecule-1 (ICAM-1) during heart ischemia. The communications between endothelial cells and these chemokines accentuate infarction-induced inflammation [117]. However, pericytes, as mural cells around vessels, protect the endothelium via interfering with adhesion molecules by forming a protective coat to reduce inflammatory activities of endothelial cells [59,158]. Overall, the inhibitory effects of pericytes on inflammatory responses blunt excessive injuries and cardiac remodeling such as fibrosis and hypertrophy in the infarcted heart.

### 5.4. Angiogenesis

Angiogenesis takes center stage in heart regeneration and cardiac repair. It was demonstrated by Payne et al. that blockage of proangiogenetic factors such as VEGF significantly reduces capillary density and impairs left-ventricle (LV) contractility [159]. The importance of angiogenesis in cardiac repair is due to all of the mechanisms involved in cardiac repair, including cardiomyogenesis, suppression of inflammatory responses, and cardiac fibrosis, which are associated with the formation of new blood vessels.

A previous study revealed that transplantation of pericytes induces the growth of capillaries, as evidenced by the increased expression of capillary marker isolectin [104]. Chen et al. found that injection of pericytes could increase numbers of pericytes not only around the infarct zone, but also inside the injured area [123]. Specifically, treatment with pericytes promotes angiogenesis in the infarcted heart mainly via the secretome. As discussed above, the pericyte is characterized as a producer of growth factors and cytokines associated with vascular remodeling during hypoxia [160]. Growth factors such as VEGF-A, PDGF-β, and TGF-β1 were found to be increased after transplantation of pericytes into the injured heart. These factors are tightly correlated to proliferation, differentiation, contractility, and stabilization of endothelial cells, suggesting the involvement of pericytes in angiogenesis via paracrine signaling [19,161,162,163,164]. Overall, the growth of new blood vessels brings nutrients and oxygen to the ischemic area, inhibits apoptosis and inflammation, and results in the eventual regeneration of the infarcted heart.

## 6. Conclusions

Currently, the role of pericytes in myocardial repair and coronary remodeling has not been defined, despite the fact that pericytes are the second most common cell type in the heart, after endothelial cells (ECs). The critical roles of pericytes in the regulation of normal blood flow and ischemia/reperfusion-induced no-reflow were identified in recent years [70,71,94]. Cardiac pericytes, as mural cells surrounding vessels, are tightly associated with adjacent cells such as endothelial cells, VSMCs, cardiomyocytes, and fibrotic cells. Pericytes and their adjacent cells are crucially involved in vascular remodeling and maintaining vascular functions, regulating blood flow, and affecting physiological and pathological processes in regeneration of the infarcted heart. Our studies, and those of other investigators, have demonstrated that cardiac pericytes can detach from the capillary and migrate into the perivascular interstitium to differentiate into myofibroblasts. This process leads to increased vascular permeability and inflammation, and it ultimately results in coronary fibrosis, perivascular fibrosis, and capillary rarefaction. This suggests that disruption of EC/PC communications occurs, thus causing pericyte detachment and promoting differentiation into myofibroblasts. Therefore, cardiac pericytes may be a novel therapeutic target for fibrosis and coronary no-reflow after myocardial infarction or ischemia/reperfusion, as well as hypertensive heart failure.

## Figures and Tables

**Table 1:** Difference between pericytes (PCs) type-I and type-II. PDGFR, platelet-derived growth factor receptor.

	Pericytes Type-I	Pericytes Type-II
Marker	PDGFR-α^+^/Nestin-GFP^−^/NG2-DsRed^+^	PDGFR-α^−^/Nestin-GFP^+^/NG2-DsRed^+^
Distribution	Capillary phenotype	Arteriolar phenotype
Function	Adipocyte deposition Fibrogenesis	Regeneration Angiogenesis

**Table 2:** Diverse shape and endothelial cell (EC) coverage ratio of pericytes and their function.

	Shape	PC-EC Ratio
Heart	Spindle-shaped	1:2–1:3
Nervous system	Solitary and stellate-shaped	1:1
Skeletal muscle		1:100
Kidney	Rounded and compact	
Function	(1) Angiogenesis and vessel stabilization (2) Capillary blood flow regulation (3) Vascular maturation and remodeling (4) Vascular permeability (5) Maintenance functional integrity of the blood–brain barrier	

**Table 3:** Molecules secreted by pericytes and their functions.

Molecules Secreted by Pericytes	Functions
TNF-α, IP-10, IL-6, 8 Eotaxin and Rantes	Induce inflammatory responses
LIF, COX-2, and HMOX-1	Inhibit inflammatory responses
Bmp-4, 6, 7	Preserve stem-cell regenerative capabilities
TGF-β	Stimulates angiogenesis and fibrogenesis
Ang-1	Enhances vessel stabilization
VEGF	Stimulates angiogenesis
SPARC	Stimulates fibrogenesis
S1P	Stabilizes intercellular contacts of PCs and ECs
OPG	Contributes to calcification
MiR-132	Induces survival response and differentiation of cardiomyocytes

Interleukin-6, 8 (IL-6, 8), tumor necrosis factor alpha (TNF-α), interferon gamma-induced protein 10 (IP-10), leukemia inhibitory factor (LIF), cyclooxygenase-2 (COX-2), heme oxygenase-1 (HMOX-1), bone morphogenetic protein-4, 6, 7 (Bmp-4, 6, 7), transforming growth factor-β (TGF-β), angiopoietins-1 (Ang-1), vascular endothelial growth factor (VEGF), secreted protein acidic and cysteine-rich (SPARC), sphingosine-1-phosphate (S1P), osteoprotegerin (OPG).

## Abbreviations

Ang-1	angiopoietins-1
α-SMA	smooth muscle α-actin
BBB	blood–brain barrier
BMP	bone morphogenetic protein
CAD	coronary arterial disease
CBF	coronary blood flow
CFR	coronary flow reserve
COX-2	cyclooxygenase-2
cTn-T	cardiac troponin-T
DLK-1	delta-like homolog 1
EC	endothelial cells
ECM	extracellular matrix
EMT	mesenchymal transition
EndMT	endothelial–mesenchymal transition
hHP	human heart pericyte
HMOX-1	heme oxygenase-1
hPSC	human pluripotent stem cell
ICAM-1	intercellular cell adhesion molecule-1
IFNγ	interferon-γ
IL	interleukin
IP-10	interferon gamma-induced protein 10
MMP	matrix metalloproteinases
OPG	osteoprotegerin
PC	pericyte
PDGFR-β	platelet-derived growth factor receptor
RGS 5	regulator of G protein signaling 5
SIRT3	Sirtuin3
S1P	sphingosine-1-phosphate
SPARC	secreted protein acidic and cysteine-rich
TGF-β	transforming growth factor-β
TIMPs	endogenous inhibitors
TNF-α	tumor necrosis factor alpha
VEGF	vascular endothelial growth factor
VSMC	vascular smooth muscle cell
VCAM-1	vascular cell adhesion molecule-1
